# Issues for Simulation of Galactic Cosmic Ray Exposures for Radiobiological Research at Ground-Based Accelerators

**DOI:** 10.3389/fonc.2015.00122

**Published:** 2015-06-04

**Authors:** Myung-Hee Y. Kim, Adam Rusek, Francis A. Cucinotta

**Affiliations:** ^1^Wyle Science, Technology and Engineering Group, Houston, TX, USA; ^2^Brookhaven National Laboratory, Upton, NY, USA; ^3^Department of Health Physics and Diagnostic Sciences, University of Nevada Las Vegas, Las Vegas, NV, USA

**Keywords:** space radiobiology, galactic cosmic rays, cancer risk, central nervous system risk, radiation transport, shielding

## Abstract

For radiobiology research on the health risks of galactic cosmic rays (GCR) ground-based accelerators have been used with mono-energetic beams of single high charge, *Z* and energy, *E* (HZE) particles. In this paper, we consider the pros and cons of a GCR reference field at a particle accelerator. At the NASA Space Radiation Laboratory (NSRL), we have proposed a GCR simulator, which implements a new rapid switching mode and higher energy beam extraction to 1.5 GeV/u, in order to integrate multiple ions into a single simulation within hours or longer for chronic exposures. After considering the GCR environment and energy limitations of NSRL, we performed extensive simulation studies using the stochastic transport code, GERMcode (GCR Event Risk Model) to define a GCR reference field using 9 HZE particle beam–energy combinations each with a unique absorber thickness to provide fragmentation and 10 or more energies of proton and ^4^He beams. The reference field is shown to well represent the charge dependence of GCR dose in several energy bins behind shielding compared to a simulated GCR environment. However, a more significant challenge for space radiobiology research is to consider chronic GCR exposure of up to 3 years in relation to simulations with animal models of human risks. We discuss issues in approaches to map important biological time scales in experimental models using ground-based simulation, with extended exposure of up to a few weeks using chronic or fractionation exposures. A kinetics model of HZE particle hit probabilities suggests that experimental simulations of several weeks will be needed to avoid high fluence rate artifacts, which places limitations on the experiments to be performed. Ultimately risk estimates are limited by theoretical understanding, and focus on improving knowledge of mechanisms and development of experimental models to improve this understanding should remain the highest priority for space radiobiology research.

## Introduction

A diverse range of health risks including cancer, central nervous system (CNS) effects, circulatory diseases, and cataracts are concerns for galactic cosmic rays (GCR) exposures during space travel (1–7). Many of these same risks are also concerns for normal tissue damage in Hadron therapy using proton and carbon beams. In this paper, we discuss the simulation of GCR for space radiobiology research with the goal of providing a new tool for risk assessment and countermeasure research and development. However, the pros and cons of GCR simulation as a tool to augment studies with single particle species need also to be addressed. The GCR environment consists of protons and high charge *Z* and energy *E* (HZE) particles with charge number, *Z* from 1 to 28, with energies from <10 MeV/u to >50 GeV/u (8–10). Of note is that nuclear fragmentation occurs in a particle accelerator beam-line due to particle passage through air and beam monitoring devices, and in the tissue of animals or cell culture dishes, media, etc. used in experiments. Heavy ion fragmentation probabilities of more than 10% occur for most experimental conditions and thus pristine mono-energetic beams do not actually occur under any circumstances. We first consider the composition of the beams to be used for a GCR simulator using multiple beam and energies combined with absorbers to provide a reference field similar to the *Z* and *E* spectrum of the GCR occurring behind typical shielding amounts inside tissue in space. In addition, the temporal dependence of biological time scales in animal or cell models used in experiments relative to the most likely durations of a deep space mission to Mars of approximately 1000 days is considered.

In considering the problem of GCR simulation, we first note that there is no single GCR environment for space missions due to several variable factors including solar cycle modulation, differences due to spacecraft material types and amounts, the shielding of the Mars atmosphere and surface albedo radiation, and variability in self-shielding of different organs, due to the variability of astronaut size and weight. The GCR are modulated over the approximately 11-year solar cycle for energies below 5 GeV/u with more than two-times higher flux at solar minimum compared to solar maximum (8, 9). There is also a 22-year periodicity in solar cycles due to shifts in our sun’s magnetic polarity (10) in successive 11-year cycles, which introduce a further GCR spectral variability. The primary energy spectra of each GCR particle species peaks at several hundred MeV/u, however, more than 50% of the GCR HZE flux is above 1500 MeV/u for typical shielding amounts (8–10). Within shielding or tissue, the energy spectra and fluence of each particle, *F*(*E, Z*), changes due to the continuous slowing down of particles in interactions with atomic electrons, and nuclear interactions leading to fragmentation and the production of new particles, including neutrons, mesons, electrons, and gamma-rays from both the GCR and target atoms. The Earth’s magnetic field shields exposures on International Space Station (ISS) missions (11) effectively blocking the primary GCR with energies below about 1 GeV/u. The surface of Mars exposures are modified by the Martian atmosphere and the albedo flux of particle produced in particle interaction with soils, while the soil composition is variable itself (12, 13). In addition, spacecraft passage through the Earth’s radiation belts and solar particle event occurrence needs to be considered making an even more diverse range of exposures. Therefore an approach for a practical solution to GCR simulation is to consider a small number of reference fields that are representative of GCR, while allowing for reproducibility for radiobiological experimentation.

The NASA radiation quality factor (QF) uses particle track structure concepts leading to a radiation quality description based on two physical parameters, particle charge number, *Z* and kinetic energy per atomic mass unit, *E*, and has replaced the use of LET due to its inaccuracy as a unique descriptor of cancer risks (9, 11). For example, relative biological effectiveness factors (RBE’s) for protons peak at LET values below 80 keV/μm while for Fe particles the RBE peak can be at an LET of 200 keV/μm or more (9, 11) as described in the NASA QF, while all particles have the same effectiveness as a function of LET in the older approaches. Importantly, biological effectiveness is predicted to decrease above 1 GeV/u for particles of approximately the same LET values due to the spreading of the particles track-width leading to larger contributions from δ-rays for relativistic particles compared to the more effective track core that is dominant for lower energy particle tracks (9, 11). For non-cancer risks, less is known about radiation quality dependence on particle type, and therefore investigations based on *Z* and *E* are also warranted.

Beyond defining reproducible reference fields, a second major consideration is the duration of chronic exposures necessary to elucidate risks in astronauts. The use of doses higher than the space condition can lead to misinformation about potential risks, especially for non-cancer effects where dose thresholds are likely and effect severity will increase with dose above a threshold. Considerations for the low GCR dose rates in space should be made in-light of the biological times scales of DNA damage processing, tissue regulation including cell turn-over in various tissues, molecular components of cognition in the CNS, and the evolution of pre-malignant cells in cancer development, etc. In this paper, we discuss how the low GCR dose rates in space lead to a straight-forward approach to chronic exposures simulation. However, the length of exposures needed to avoid dose-rate artifacts will make a true simulation exceedingly costly.

The GCR simulator being developed at the NASA Space Radiation Laboratory (NSRL) located at Brookhaven National Laboratory (BNL) (14) was conceived by one of the present authors (Francis A. Cucinotta) in 2008 (15) during the development of the new BNL electron beam injector source (EBIS) for use at the NSRL and the BNL Relativistic Heavy Ion Collider (RHIC) (16). The upgrade includes a rapid beam switching mode of about 1-min intervals over multiple ion sources, and the addition of new power sources to allow higher beam energies up to 1.5 GeV/u for HZE particles and 4 GeV for protons compared to the current maximum (1.0 GeV/u for HZE particles and 2.5 GeV for protons). In this paper, we consider the design of GCR simulator at the NSRL and recommend a reference field defined by a GCR *Z*-spectrum in major energy bins that matches validated predictions of space radiation environments (12, 13, 17) for two specific shielding configurations. Pros and cons for space radiobiology research are then discussed and shown to limit the usefulness of a GCR simulator to a narrow range of research questions.

## Materials and Methods

We focus on the development of a GCR simulator for the near solar minimum environment because of its higher concern for risk assessments. A second variable to consider is the amount and types of spacecraft shielding and a representative tissue self-shielding. Organ doses and dose equivalents show small variation from GCR, and we therefore considered simulating the average tissue as represented by 5-cm tissue equivalent shielding often used to represent the blood forming organ (BFO) self-shielding distribution (8, 18). We note that experiments with small animals such as mice or rats along with holders, where animals are placed, lead to an additional 2–10 cm of tissue equivalent shielding, such that the use of 5-cm tissue equivalent shielding and these additions result in a simulation that accurately represents the average organ depth in humans. Target fragments produced from tissue atoms (19) will be simulated accurately at such depths of tissue because of the dominance of short-ranged proton and helium fragments produced locally from constituent atoms. We consider the typical spacecraft shielding thickness, which with internal equipment a thickness of about 20 g/cm^2^ aluminum equivalent, while a minimum shielding of 5 g/cm^2^ occurs. These two shielding configurations are denoted as the transfer vehicle and surface habitat. In the present paper, we did not consider simulation of exposures on the Mars surface, however this area will be considered in future work. For the Mars surface environment, the energy spectra of neutrons will be an important factor as described in our recent papers (12, 13).

### Validated space environment prediction

In order to estimate the space environment within spacecraft shielding, a large number of spaceflight measurements are considered and used to validate computer code predictions. The representation of the GCR particle distribution consists of the free space environment, radiation transport model, and shielding distribution. Extensive spectral measurements of particle type and energy distributons have been reported from satellite and baloon experiments in Antartica using large instruments (>10 kg) that are typically not used on human missions. These data have been used to formulate an accurate computer model of the free space GCR for particles from protons to nickel particles for energies from 0 to 50 GeV/u (10, 20). For the calculations in this report, we use the GCR environment model at 1977 solar minimum. As a model of the GCR environment behind shielding, we use the high-charge and energy (HZETRN) transport code (18, 21, 22), which solves for the spectrum of nuclear fragments from projectile and target nuclei in the continuous slowing down and straight-ahead approximation. The HZETRN code has been compared to extensive flight measurements for dose and dose equivalent on space shuttle, ISS, and Mars transit and Mars surface measurements and generally agree with these measurements to within ±15% (9, 12, 13, 17).

For the nuclear interactions of the primary GCR with the matter, the quantum multiple scattering theory of nuclear fragmentation (QMSFRG) model describes the production of light nuclei through the distinct mechanisms of nuclear abrasion and ablation, coalescence, and cluster knockout (22, 23). Helium interaction cross sections were described previously by Cucinotta et al. (24, 25) while the HZETRN code uses proton and neutron interaction cross sections from the Ranft and Bertini models of nuclear cascade and evaporation processes (18, 21).

### Reference fields at NSRL

In the design of GCR reference field, the changes in the beam composition or energy behind proposed absorbers due to energy loss and fragmentation and production of secondary radiation by the absorber are simulated using the GERMcode (26). For the design of a reproducable reference field, we consider a configuration with a small number of ion sources: p, ^4^He, ^16^O, ^28^Si, and ^56^Fe. Energy switching is then considered with possible absorbers to spread both the energy and fragment distribution to represent the GCR with some realistic measure in specific Z and E bins. Three energy changes each for ^16^O, ^28^Si, and ^56^Fe are considered, and additional energy changes for p and ^4^He beams, as described next. The use of a computer-controlled automated binary filter is assumed to allow for beam-specific variable absorber amounts to optimize the spreading and fragmentation of the beam for the purpose of obtaining the desired *Z* and *E* dependence of particles at the biological samples. The thickness of the absorber is chosen to reproduce the HZETRN code results for the *Z*-dependence of particle absorbed dose in the energy bins considered. Particles of lower energy (<50 MeV/u) are a minimal consideration for several reasons, including their stopping in absorbers or the entrance tissues of animals, the continuous slowing down of higher energy particles in the absorber will produce particles following a characteristic 1/LET(*E*) spectral shape (18) within tissues, and lower energy particles are produced locally in nuclear absorption events of high energy particles.

#### Light Ions

The NSRL can provide energies of protons and ^4^He ranging from as low as 40 MeV/u to about 2.5 and 1 GeV/u currently and up to 4 and 1.5 GeV/u, respectively, with the proposed NSRL energy upgrade. In the design of broad energy range of protons and helium comprising the most abundant in GCR, the beam fluence of a specified energy bin is calculated from the dose and fluence relation,

(1) D=ρΦL

where, ρ is the density of material, Φ is the number of particles per unit area, fluence, and *L* is the rate of energy loss, LET. We consider a number of energy changes, 10 or more, for both proton and ^4^He beams with the precise number considered in design tests of energy resolution relative to experimental simulation times. The QF or RBE for the *Z* = 1 and 2 particles above a few hundred MeV/u will be close to unity and largely independent of energy making the energy resolution a minor consideration for biological responses. Other H and He isotopes will be produced in the reference field due to projectile and target fragmentation of the various beam–target interactions that result from the overall simulation. The secondary mesons produced through multi-particle production processes at the highest energies (from 3 to 4 GeV) better represent the space situation compared to lower energy particles (<1 GeV/u) where pion production is dominated by one pion production cross-sections. However, these differences will not be significant for biological responses because high-energy pions and their decay products have low RBE, especially for shielding amounts below 100 g/cm^2^.

#### HZE Particles

In order to accurately simulate the GCR charge and energy distribution of dose, three major HZE beams (^16^O, ^28^Si, and ^56^Fe) are selected at three energies (500, 900, and 1500 MeV/u). By placing the absorber of polyethylene, the primary particles will interact with the absorber losing energy and producing secondary nuclei through projectile fragmentation. The absorber thickness is chosen to match the *Z*-distribution predicted by the HZETRN code in three energy bins (0–500, 500–900, and >900 MeV/u). Here, an initial estimate of the absorber thickness of a specified primary ion at the energy bin is calculated from the absorption and extinction rate as,

(2) $$\frac{D_{\text{j}}(E_{\text{bin}})}{D_{Z\text{\_group}}(\;E_{\text{bin}})}=e^{(-\sigma_{\text{j}}\left(E\right)x_{\text{pe}}\text{)}}\;$$

where, *D*_j_(*E_bin_*) is the dose of the primary j ion in the energy bin (j for ^16^O, ^28^Si, and ^56^Fe; *E*_bin_ for *E* < 500 MeV/u, *E* = 500–900 MeV/u, or *E* > 900 MeV/u), *D_Z_*__group_(*E*_bin_) is the total dose of the corresponding charge group in the energy bin for j (*Z*_group for *Z* = 3–8, *Z* = 9–14, or *Z* = 15–28), σ_j_(*E*) is the macroscopic absorption cross-section for the primary j with energy *E*, and *x*_pe_ is the depth of polyethylene absorber.

The dose without the absorber relative to those with the absorber is related to the primary beam and its fragments, respectively. Particle fluence is calculated using the GERMcode (26) for the primary ion without the absorber in Eq. 3a and for the beam fluence required to obtain the dose from fragmentation and energy loss by the absorber in Eq. 3b.

(3a) $$\Phi_{\text{j}}(E,x_{0})=D_{\text{(}Z_{\text{group}}\text{)}}(E_{b}in)\text{×}B_{\text{(abs, j)(}E\text{)}}\text{×}\Phi_{\text{j}}(E){\text{Φ}}_{({\text{Gy-μm}}^{\text{2}}\text{)}}$$

(3b) $$\Phi_{\text{j}}(E,x_{\text{pe}})=D_{Z\text{\_group}}\left(E_{\text{bin}}\right)\times \;(1.-B_{\text{abs, j}}(E))\;\times \;\Phi_{\text{j}}{\left(E\right)}_{{\text{Gy-μm}}^{\text{2}}}\times \frac{D_{\text{j}}(E,x_{0})}{D_{\text{j}}(E,x_{\text{pe}})}$$

Here, *D*_Z_group_ (*E*_bin_) are the dosimetric quantities predicted from the HZETRN code. *B*_abs, j_ (*E*) is the beam absorption rate of the primary j ion at energy *E* as calculated in Eq. 2. The beam fluence of j ion at energy *E* to the area of 1 μm^2^ for the exposure to 1 Gy, and the dose without absorber to that with the absorber, can be obtained from the GERMcode (26).

### Duration and order of exposures

We next consider the proposed beam-energy combination described above and NSRL capability for inter-fraction time for mixed sources as short as 1 min, in order to recommend time profiles for GCR simulation. To obtain reasonable statistics 10 exposures each for our preliminary beam-energy combinations or ~350 fractions are considered as a first estimate resulting in a GCR simulation of about 6 h. When considering the total annual GCR dose of ~200 mGy/y, we note that a small number of particles per pulse has been used previously at NSRL with no technical issues (27). A more refined estimate considers the dose weighting required for different beams such that a higher number of proton and helium pulses compared to the O, Si, and Fe beams. In a shift from proton to proton, or proton to helium, exposures of different energies will occur frequently under these conditions, and therefore we estimate about 8–10 h-exposure duration would provide a reasonable simulation time based on beam delivery and dose weighting considerations alone. However, biological response time scales, including the kinetics of responses in experimental models compared to astronauts on space missions, lead to further considerations as described next.

We considered a kinetics formalism of the multi-hit model to estimate the number of cells hit by HZE particles during the evolution of a chronic exposure. The model assumes the particle hits are Poisson distributed. We consider the fluence rates for HZE particles alone or including protons and helium, and estimates of cell or tissue structure sensitive areas, *A*, with biological processes relaxation times, τ_relax_, such as DNA repair or signal transduction. The mean hit-rate per day, *H*_r_, is estimated using *A* and the fluence rate, *F* by

(4) $$H_{\text{r}\;}=FA$$

The kinetics representation of the Poisson distribution of cells (or sensitive tissue areas) with 0, 1, 2, etc. hits denoted as *n_i_* at any given time is given by the system of ordinary differential equations:

(5a) $$\begin{array}{cccc} & \frac{\text{d}n_{\text{o}}(t)}{\text{d}t} & = & -H_{\text{r}}n_{\text{0}}(t)+K_{\text{D}} \end{array}\sum_{i\text{=1}}\begin{array}{cc} f_{i} & n_{i}(t) \end{array}$$

(5b) $$\begin{array}{cccc} & \frac{\text{d}n_{i}(t)}{\text{d}t} & = & H_{\text{r}}n_{i\text{-1}}^{}(t)-(H_{\text{r}}{}_{}+K_{\text{D}}+K{\left(i\right)}_{\text{in}}) \end{array}n_{i}(t)$$

Where *K*_D_ is the decay rate given by ln (2)/τ_relax_, *K*(*i*)_in_ is rate of cell death after the *ith*-hit, and *f_i_* is the fraction of *n_i_* cells that are not eliminated by the *ith*-hit. Using hit rates from the GERMcode simulations described in this report and several possible relaxation times, Eqs (5a) and (5b) are solved numerically to make predictions comparing these cell populations for chronic exposures of varying lengths.

The order of exposures in space is random as weighted by particle fluence for a given species and energy. Using random number generators, a random order of exposures can easily be obtained. For biological research replicate experiments are required, such that a computer model generated order should be used for each replicate experiment. Selecting a different order with proper weighting would not likely change the experimental results within expected uncertainties, however may require further considerations.

## Results

**Figure 1:**
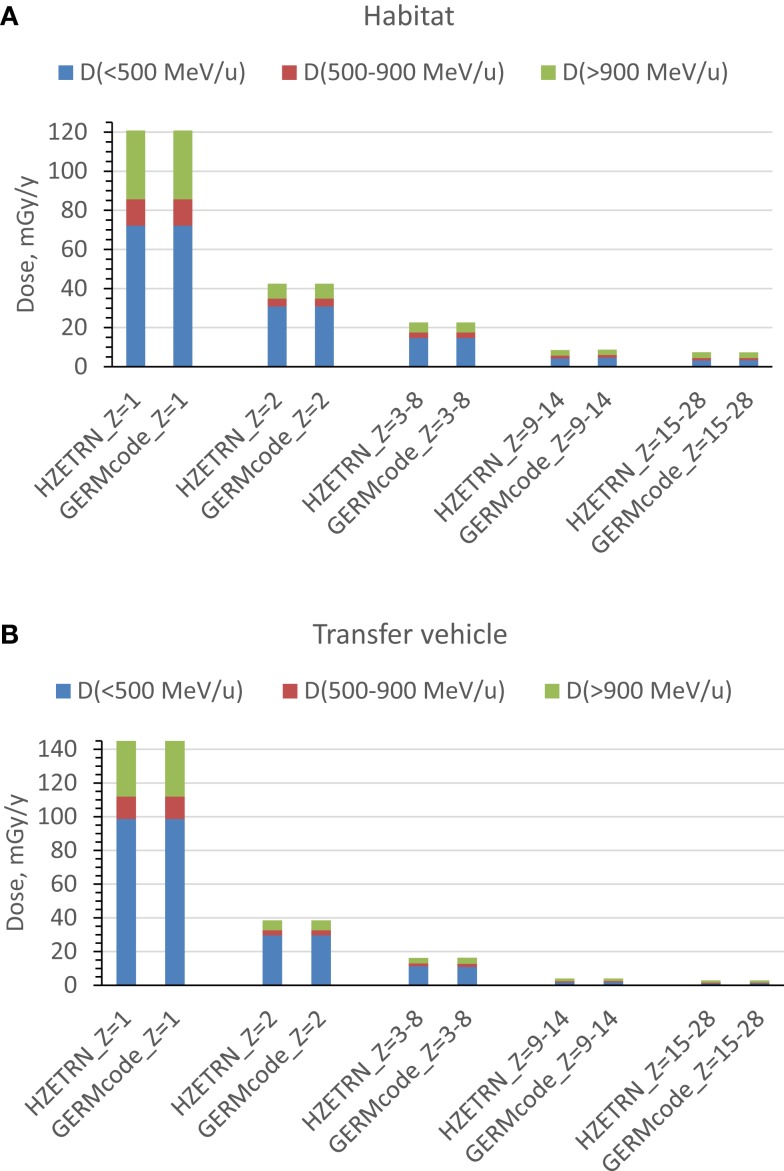
**Prediction of annual 5-cm tissue equivalent dose for energy and charge groups from exposure to annual GCR at 1977 solar minimum**. **(A)** habitat, **(B)** transfer vehicle.

Two spherical configurations of 20 and 5 g/cm^2^-thick aluminum are used for an equivalent Mars transfer vehicle and the minimum amount for pressure vessel-wall in living quarter, respectively. The annual 5-cm tissue doses from exposure to GCR at 1977 solar minimum environment are simulated after passing through shielding configurations of a Mars transfer vehicle or a habitat. The *Z*-group dependence of the dose from the prediction of HZETRN code for the two shielding configurations are reported in Tables 1 and 2 and Figure 1. These results show the expected dominance of protons and helium to tissue doses for typical shielding amounts. Table 3 shows the HZETRN predictions of the energy spectra for hydrogen and helium particles for the two shielding configurations in different energy bins. The highest energy bin includes integral contributions from all particles above this energy. The light ion beam fluence per unit area at each energy is also shown in Table 3, which is calculated with regard to the corresponding dose fraction of light ions in each energy bin. Not shown are the variation of the spectra at lower energies where hydrogen and helium particles reach high LET values (>10 keV/μm); however these particles follow a characteristic spectra of 1/LET(*E*) from atomic slowing down or are produced locally due to their small range (18). Thus they are adequately represented by the use of the 5-cm tissue equivalent shielding along with the additional materials of the tissue for the animal model considered.

**Table 1:** **Contributions from different charge groups predicted by HZETRN code for two reference shielding designs**.

Charge group, *Z*	Habitat + 5-cm tissue		Transfer vehicle + 5-cm tissue	
	Dose fraction	Dose, mGy/y	Dose fraction	Dose, mGy/y
*Z* = 1	0.60	120.8	0.70	145.0
*Z* = 2	0.21	42.5	0.19	38.5
3 ≤ *Z* ≤ 8	0.11	22.7	0.08	16.3
9 ≤ *Z* ≤ 14	0.04	8.5	0.02	4.0
15 ≤ *Z* ≤ 28	0.04	7.4	0.01	2.9
Sum	1.00	201.9	1.00	206.7

**Table 2:** **Comparison of doses of several charge (*Z*)-groups in three energy bins from the HZETRN code to the model GCR reference field (in parenthesis)**.

	Habitat + 5-cm tissue			Transfer vehicle + 5-cm tissue		
	Dose, mGy			Dose, mGy		
*E*, MeV/u	<500	500–900	>900	<500	500–900	>900
*Z* = 3–8	14.7 (14.2)	2.9 (3.0)	5.2 (5.6)	11.3 (10.9)	1.7 (1.8)	3.3 (3.6)
*Z* = 9–14	4.3 (4.6)	1.4 (1.4)	2.9 (2.7)	1.7 (1.8)	0.7 (0.7)	1.6 (1.5)
*Z* = 15–28	3.3 (3.3)	1.2 (1.2)	2.9 (2.9)	1.1 (1.1)	0.5 (0.5)	1.4 (1.3)

**Table 3:** **Beam energy and fluence for light ions (*Z***=** 1 and 2)**.

Protons			^4^He		
*E_p_*, MeV	*D*_p_, mGy	Φ_p_/μm^2^	*E*_He_, MeV/u	*D*_He_, *mGy*	Φ_He_/μm^2^
**(A) INSIDE THE HABITAT (5 g/cm^2^ ALUMINUM **+** 5-cm TISSUE)**					
50	28.05	1.39 × 10^−1^	50	14.54	1.80 × 10^−2^
100	12.34	1.05 × 10^−1^	100	4.42	9.36 × 10^−3^
200	13.11	1.81 × 10^−1^	200	4.86	1.68 × 10^−2^
300	7.91	1.39 × 10^−1^	300	3.07	1.35 × 10^−2^
400	5.93	1.04 × 10^−1^	400	2.23	1.14 × 10^−2^
500	4.85	1.09 × 10^−1^	500	1.69	9.52 × 10^−3^
600	4.1	9.97 × 10^−2^	600	1.32	7.98 × 10^−3^
800	6.63	1.76 × 10^−1^	800	1.95	1.30 × 10^−2^
900	2.72	7.46 × 10^−2^	900	0.75	5.14 × 10^−3^
1000	2.42	6.79 × 10^−2^	1000	0.63	4.43 × 10^−3^
1200	4.09	1.18 × 10^−1^	1200	7.01	5.08 × 10^−2^
1400	3.31	9.79 × 10^−2^			
1600	2.72	8.13 × 10^−2^			
1800	2.27	6.85 × 10^−2^			
2000	1.92	5.81 × 10^−2^			
2500	18.46	5.59 × 10^−1^			
Total	120.8		Total	42.47	
**(b) INSIDE THE TRANSFER VEHICLE (20 g/cm^2^ ALUMINUM **+** 5-cm TISSUE)**					
50	40.28	2.00 × 10^−1^	50	15.92	1.97 × 10^−2^
100	18.58	1.58 × 10^−1^	100	4.21	8.93 × 10^−3^
200	18.61	2.57 × 10^−1^	200	4.09	1.41 × 10^−2^
300	9.71	1.71 × 10^−1^	300	2.37	1.04 × 10^−2^
400	6.53	1.15 × 10^−1^	400	1.69	8.64 × 10^−3^
500	5.02	1.14 × 10^−1^	500	1.27	7.17 × 10^−3^
600	4.12	1.00 × 10^−1^	600	0.99	6.00 × 10^−3^
800	6.5	1.73 × 10^−1^	800	1.5	1.00 × 10^−2^
900	2.63	7.20 × 10^−2^	900	0.6	4.09 × 10^−3^
1000	2.32	6.51 × 10^−2^	1000	0.5	3.49 × 10^−3^
1200	3.89	1.13 × 10^−1^	1200	5.38	3.90 × 10^−2^
1400	3.13	9.26 × 10^−2^			
1600	2.56	7.67 × 10^−2^			
1800	2.13	6.45 × 10^−2^			
2000	1.8	5.46 × 10^−2^			
2500	17.21	5.21 × 10^−1^			
Total	145		Total	38.5	

**Figure 2:**
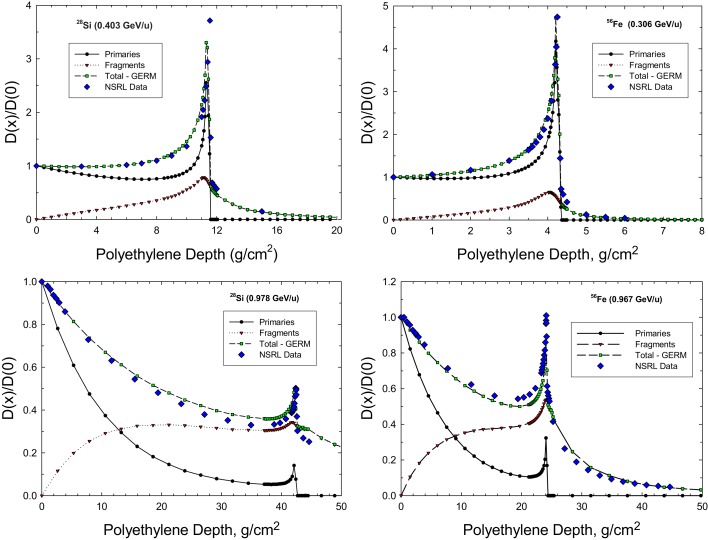
**Comparison of GERMcode and NSRL measurements for the depth-dose distribution in polyethylene shielding for ^28^Si and ^56^Fe beams**.

Figure 2 shows comparisons on the GERMcode simulation to measurements at NSRL for Bragg curves in polyethylene for beams ^28^Si at 0.4 and 0.98 GeV/u and ^56^Fe at 0.3 and 0.97 GeV/u. The GERMcode accurately predicts the depth-dose distribution, and predicts the so-called tail distribution of fragments that go well beyond the range of the primary beam. As the kinetic energy and projectile beam mass increases, the Bragg peak is diminished and a nearly exponential depth-dose distribution will occur above a few GeV/u.

Table 4 shows the extinction fraction of the specified heavy ions of a GCR simulator needed to match the prediction of dose fraction of GCR ion from HZETRN code, and the prediction of the depth of polyethylene absorber according to Eq. (2) with the macroscopic absorption cross sections of the ions in polyethylene, σ_abs_. In the design of a GCR simulator, the dose distribution from particles with charge numbers other than primary beam is through fragmentation of the selected heavy ion. Tables 5 and 6 show the modified absorber depth to promote more fragments from high energy beams, by which the dose fraction of *Z*-group at each energy bin has been best matched to the prediction of HZETRN code. Tables 5 and 6 also show the mean energy of the primary beam after penetrating the absorber distance. In the current design, the primary beam does not completely stop after the absorber depth (except 500 MeV/u ^28^Si) due to the downgraded energy of the beam. A broad energy range behind the absorber depth results for the projectiles and fragments. The corresponding heavy ion beam fluence of the primary and the fragments at 5-cm tissue inside the habitat and the transfer vehicle are also shown in Tables 5 and 6, respectively.

**Table 4:** **Heavy ion beam extinction fraction for GCR simulator based on dose fraction of the beam predicted from the HZETRN code, absorber depth in polyethylene *x*_pe_, for beam extinction fraction, and macroscopic absoprtion cross sections, **σ**_abs_, of the beam in polyethylene**.

HZE beam		GCR dose fraction of the ion	Habitat + 5 cm tissue		Transfer vehicle + 5-cm tissue		σ_abs_, cm^2^/g
			Extinction	*x*_pe_, g/cm^2^	Extinction	*x*_pe_, g/cm^2^	
500 MeV/u	^16^O	D_16O_/Σ(D_3–8_)	0.305	17.8	0.169	26.6	0.0668
	^28^Si	D_28Si_/Σ(D_9–14_)	0.299	13.2	0.236	15.7	0.0916
	^56^Fe	D_56Fe_/Σ(D_15–28_)	0.410	6.8	0.309	9.0	0.1307
900 MeV/u	^16^O	D_16O_/Σ(D_3–8_)	0.447	18.0	0.382	20.5	0.0706
	^28^Si	D_28Si_/Σ(D_9–14_)	0.306	21.5	0.250	23.5	0.0966
	^56^Fe	D_56Fe_/Σ(D_15–28_)	0.430	8.2	0.327	12.9	0.1366
1500 MeV/u	^16^O	D_16O_/Σ(D_3–8_)	0.455	17.2	0.396	19.5	0.0722
	^28^Si	D_28Si_/Σ(D_9–14_)	0.290	21.5	0.240	24.0	0.0987
	^56^Fe	D_56Fe_/Σ(D_15–28_)	0.455	7.4	0.350	12.0	0.1392

**Table 5:** **Heavy ion beam fluence in energy bin with polyethylene absorber for mixed-field spectrum inside the habitat (5 g/cm^2^ aluminum **+** 5-cm tissue) to match the dose of *Z*-group at energy bin predicted from HZETRN code. The beam energy and average energy of the beam after the absorbor, *E*_out_. are shown**.

j	*E*, MeV/u	*x*_pe_, g/cm^2^	*E*_out_, Me V/u	$\frac{D_{\text{j}}(E,x_{\text{pe}})}{D_{\text{j}}(E,x_{0})}$^a^	Primary or fragments	*D_j_*(*E*), mGy^b^	Φ_j_(*E*)/μm^2^/Gy^a^	Φ_j_(*E*)/μm^2^
^16^O	500	0		1	0.164	2.25	0.353	7.94 × 10^−4^
		17.8	119	0.86	0.836	11.48		4.69 × 10^−3^
	900	0		1	0.271	0.73	0.428	3.12 × 10^−4^
		18	516	0.64	0.729	1.96		1.30 × 10^−3^
	1500	0		1	0.282	1.36	0.465	6.34 × 10^−4^
		17.2	1127	0.61	0.718	3.48		2.64 × 10^−3^
^28^Si	500	0		1	0.151	0.74	0.115	8.50 × 10^−5^
		13.2	52	0.99	0.849	4.16		4.84 × 10^−4^
	900	0		1	0.206	0.32	0.140	4.53 × 10^−5^
		21.5	286	0.48	0.794	1.25		3.61 × 10^−4^
	1500	0		1	0.207	0.68	0.152	1.03 × 10^−4^
		21.5	886	0.4	0.793	2.61		9.91 × 10^−4^
^56^Fe	500	0		1	0.207	0.69	0.034	2.33 × 10^−5^
		6.8	85	0.98	0.793	2.65		9.03 × 10^−5^
	900	0		1	0.256	0.33	0.041	1.32 × 10^−5^
		8.2	433	0.67	0.744	0.95		5.70 × 10^−5^
	1500	0		1	0.276	0.83	0.044	3.66 × 10^−5^
		7.4	1062	0.65	0.724	2.18		1.48 × 10^−4^

*^a^The relative dose behind the absorber and the beam fluence to 1 μm^2^/Gy from GERMcode*.

*^b^Based on the *Z*-group of dose from the HZETRN prediction*.

**Table 6:** **Heavy ion beam fluence in energy bin with polyethylene absorber for mixed-field spectrum inside the transfer vehicle (20 g/cm^2^ aluminum **+** 5-cm tissue) to match the dose of *Z*-group at energy bin predicted from HZETRN code. The beam energy and average energy of the beam after the absorbor, *E*_out_ are shown**.

j	*E*, MeV/u	*x*_pe_, g/cm^2^	*E*_out_, MeV/u	$\frac{D_{\text{j}}(E,x_{\text{pe}})}{D_{\text{j}}(E,x_{0})}$^a^	Primary or fragments	*D*_j_(*E*), *m*Gy^b^	Φ_j_(*E*)/μm^2^/Gy^b^	Φ_j_(*E*)/μm^2^
^16^O	500	0		1	0.076	0.82	0.353	2.89 × 10^−4^
		26.6	10	1.22	0.924	9.9		2.86 × 10^−3^
	900	0		1	0.237	0.38	0.428	1.61 × 10^−4^
		20.5	484	0.61	0.763	1.21		8.48 × 10^−4^
	1500	0		1	0.252	0.79	0.465	3.68 × 10^−4^
		19.5	1097	0.57	0.748	2.35		1.90 × 10^−3^
^28^Si	500	0		1	0.096	0.19	0.115	2.24 × 10^−5^
		15.7	0	1.4	0.904	1.82		1.50 × 10^−4^
	900	0		1	0.17	0.13	0.140	1.84 × 10^−5^
		23.5	249	0.46	0.83	0.64		1.94 × 10^−4^
	1500	0		1	0.176	0.33	0.152	4.96 × 10^−5^
		24	839	0.37	0.824	1.53		6.37 × 10^−4^
^56^Fe	500	0		1	0.136	0.15	0.034	4.98 × 10^−6^
		9	4	1.3	0.864	0.94		2.43 × 10^−5^
	900	0		1	0.208	0.1	0.041	4.11 × 10^−6^
		12.9	273	0.57	0.792	0.39		2.76 × 10^−5^
	1500	0		1	0.233	0.33	0.044	1.44 × 10^−5^
		12	902	0.5	0.767	1.08		9.45 × 10^−5^

*^a^Relative dose behind the absorber and beam fluence to 1 μm^2^/Gy from GERMcode*.

*^b^Based on the *Z*-group of dose from the HZETRN prediction*.

**Figure 3:**
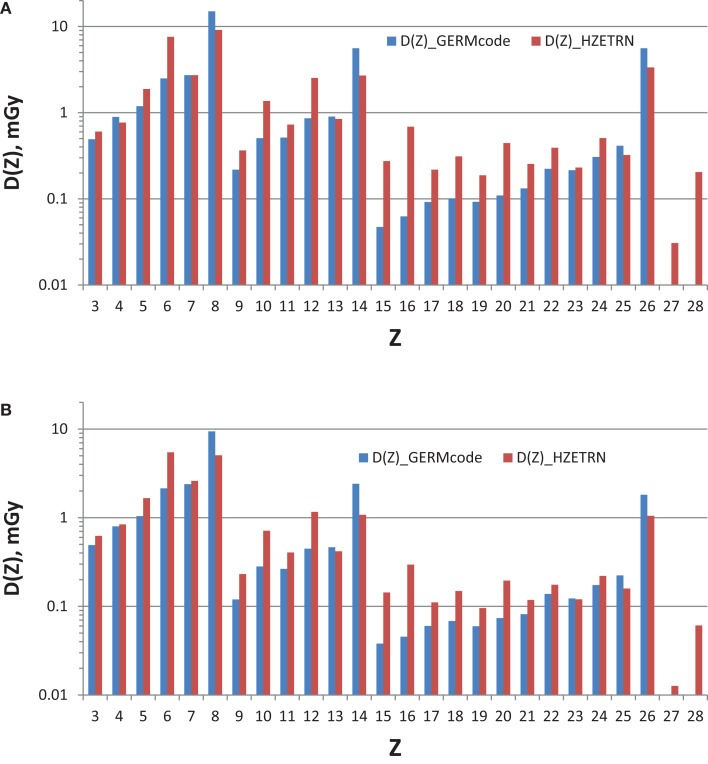
***Z*-distribution of dose at 5-cm tissue by the GCR reference field using nine HZE beam-energy combinations compared to those by the simulated full GCR spectrum at 1977 solar minimum inside habitat wall (A) and transfer vehicle (B)**.

**Figure 4:**
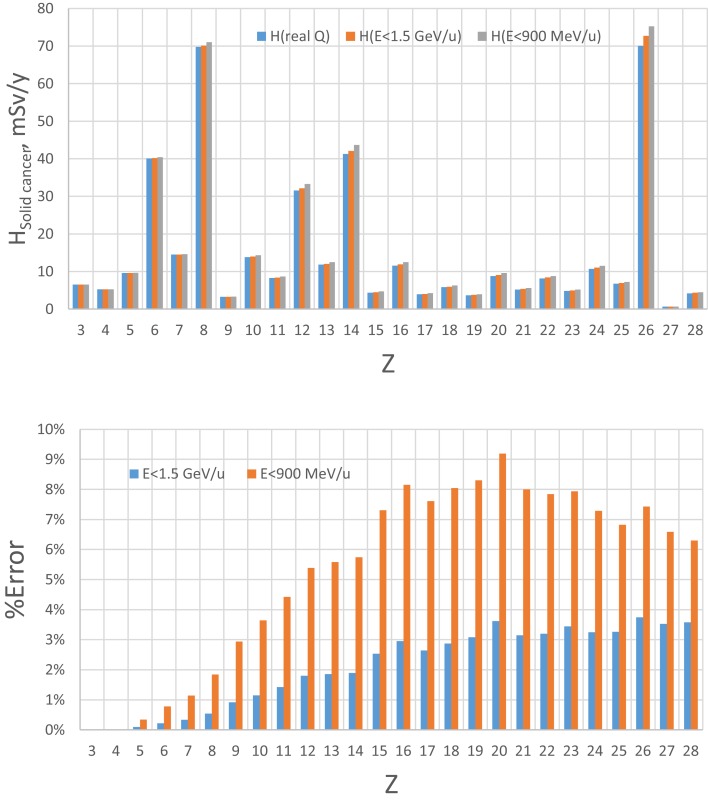
**Estimate of error of GCR simulator by accelerator energy cutoff of 1.5 GeV/u or 900 MeV/u for solid cancer risk at 5-cm tissue equivalent depth inside habitat wall from exposure to annual GCR at 1977 solar minimum**.

The current GCR reference field using nine HZE beam-energy combinations with absorber is compared to simulated full GCR environments in terms of *Z*-group dose distribution in Table 2, where good agreement is found. The individual charge contributions to the dose distribution are shown in Figures 3 and 4. Improved matching of several GCR elements such as *Z* = 6, 10, and 12 will require the use of a larger number of primary beams. To minimize the error of overall *Z*-distribution of dose and to best match the *Z*-group dose as shown in Table 2, the required doses of the primary beams are listed in Table 6, *D*_j_(*E*). The resultant dose distribution in Figures 3 and 4 shows that our minimal GCR reference field describes qualitatively very well the representative *Z*-distribution of dose for full simulated GCR environment, and can be easily improved by the use of a larger number of primary beams.

**Figure 5:**
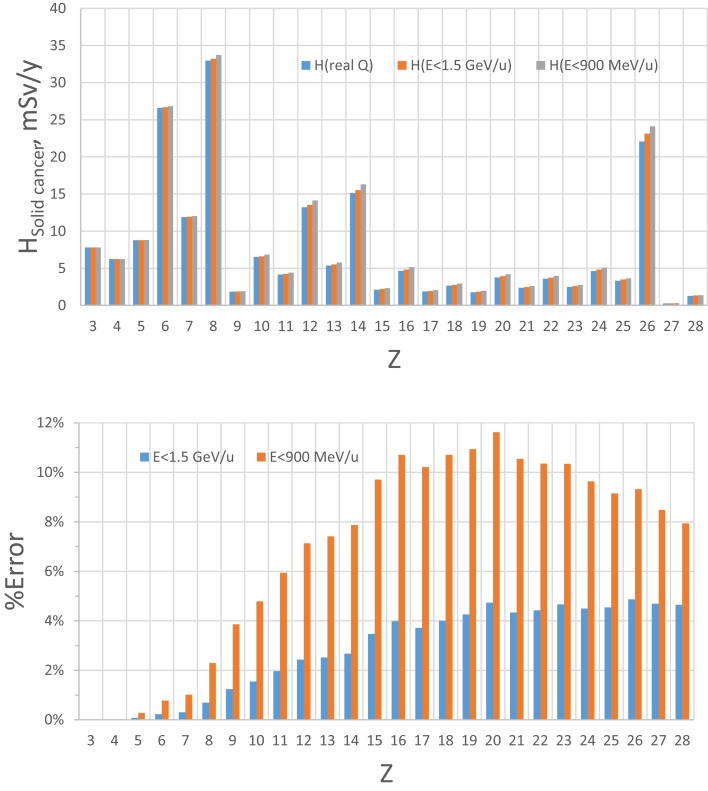
**Estimate of error of GCR simulator by accelerator energy cutoff of 1.5 GeV/u or 900 MeV/u for solid cancer risk at 5-cm tissue equivalent depth inside transfer vehicle from exposure to annual GCR at 1977 solar minimum**.

The limitation of upper energy of the simulation compared to the GCR environment in space introduces some error because the dose above 1.5 GeV/u (4 GeV) for HZE particles (protons) is assumed to be represented by the upper energy bin as described above. The magnitude of this error will depend on which biological response is considered. Based on the central estimates of the NASA QF function for solid cancer risk (9), we estimated the error by using the HZETRN code, for which predictions with the QF held fixed at either 900 or 1500 MeV/u are compared to those using the actual energy dependence in the NASA QF values (Figures 4 and 5 for 5 and 20 g/cm^2^, respectively). The GCR simulator overestimates the dose equivalent because the NASA QF decreases above the cutoff energy. This is in contrast to the older QF models used by the International Commission on Radiological Protection (ICRP) for ground-based exposures where the LET dependence is such that QF increased with increasing energy above 1.5 GeV/u for most GCR heavy ions. The error using the NASA solid cancer QF basis is quite reasonable being <12 or 5% for a cutoff of 900 or 1500 MeV/u, respectively. It is important to emphasis the higher energies (>1 GeV/u) are needed at an accelerator to obtain particles of sufficient depth of penetration in a mixed-field as well as minimizing the error in dose equivalent simulations.

It is well known that for HZE particle fluence in space each cell nucleus would receive only 0 or 1 particle traversal with nearly 100% probability, such that there is a negligible probability for individual cells to receive two or more HZE particle hits on long-term space missions. Larger targets suggested by non-targeted effects or for damage to neuron cells including dendritic trees (4) lead to other considerations. At the other extreme, each cell will receive about 1 particle traversal per 2 days for proton fluence near solar minimum and about an equal number of δ-ray cell traversal (28). If a major consideration was about protons or δ-rays hitting the same target cell within the simulated time compared to the space condition of about one per day, then a minimal GCR simulation time of at least 2 days should be utilized.

**Figure 6:**
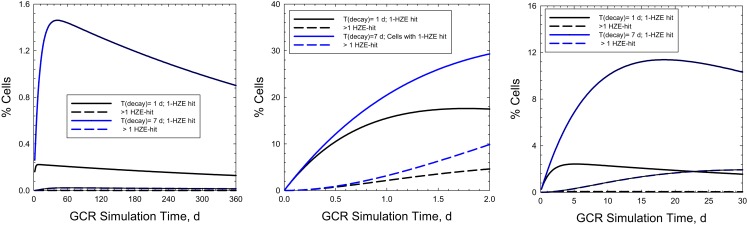
**Predictions of percentage of cells with 1 or **>**1 HZE particle hits as a function of exposure time in chronic irradiations with 20 g/cm^2^ aluminum and 5-cm tissue shielding**. Results for a 1-year mission **(A)** are compared to 30-days and 2-days ground-based simulations **(B,C)** for biological response relaxation times of 1- or 7-days assuming cell areas of 100 μm^2^.

**Figure 7:**
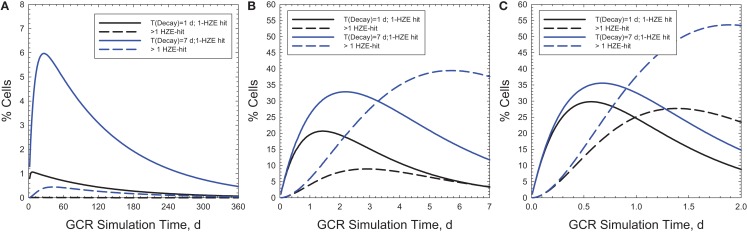
**Predictions of percentage of cells with 1 or **>**1 HZE particle hits as a function of exposure time in chronic irradiations with 20 g/cm^2^ aluminum and 5-cm tissue shielding**. Results for a 1-year mission **(A)** are compared to 30- and 2-days ground-based simulations **(B,C)** for biological response relaxation times of 1 or 7-day assuming cell areas of 500 μm^2^.

Figure 6 shows the time-dependent probabilities for cells with 100 μm^2^ area to receive 1 or >1 particle hit at a given time point during the actual GCR exposure over 1-year (Figure 6A) or NSRL simulations of 30 or 2 days (Figures 6B,C, respectively). Similar comparisons are shown in Figure 7 for a larger area of 500 μm^2^. Predictions for relaxation times of 1 or 7 days are shown assuming an average rate of cell inactivation of 10% per hit (29). For example for a 2 day, GCR simulation assuming a 100 μm^2^ target size 5 or 10% of cells will receive two or more HZE hits for relaxation times of 1 or 7 days, respectively. Larger multi-hit percentages occur for the 500 μm^2^ where the two or more HZE particle hit probability exceeds the one-hit probability for a 2 or 30 days simulation. This larger area would be more representative of areas suggested by non-targeted effects studies (30, 31) or neuronal cell structures (7). Cells with multiple hits will likely have a significantly higher response compared to cells with a single HZE traversal and thus could dominate responses, and short (<1 week) exposure times will likely lead to over-estimation of effect. Multiple-hit artifact contributions will increase for shorter simulation times. However, the beam-time costs and limitations in types of endpoints to be observed in experiments of this duration are large hurdles in using a GCR simulation for improving risks models and reducing their uncertainties.

## Discussion

The transport code predictions discussed in this paper suggest that very detailed simulations of the *Z* and *E* dependence of HZE particle doses can be made with only a few beam type and energy changes using an automated absorber depth for each primary beam. Based on the current technologies at the NSRL GCR simulations within 8 h would be possible but would not be representative of the space situation because of the multiple-hits per cell or neuronal structure artifacts that would arise. GCR simulations based on particle charge and energy are needed due to the inaccuracy of LET as a descriptor for both cancer (9) and CNS effects (30). The error introduced by an HZE particle cut-off of 1500 MeV/u relative to the particle spectrum in space is small for the solid cancer dose equivalent, however the energy upgrade at NSRL is needed to obtain particles of significant range to represent spacecraft or planetary atmosphere shielding. Errors introduced for CNS or other risk estimates have not been evaluated and would be difficult to estimate based on the limitations in current CNS animal data (4).

High energy protons and helium particles are of low RBE and simulation of the details of their energy spectra above 100 MeV/u is not critical and can be considered in terms of their cumulative doses and target fragment production. However, a large number of energy changes for these beams should be possible based on previous exposures simulating solar particle events at NSRL. Neutrons are produced in the absorbers or tissue equivalent materials using our design through nuclear reactions. Additional neutrons are produced in tissues of mice or rats and holders to be employed in experiments. Low energy neutrons (<5 MeV) are known to have large RBEs for late effects and their dose contributions will be reproduced accurately if the high-energy charged particle composition and energy spectra are simulated accurately. Protons and helium particles create the most neutrons in space because of their much higher fluence amongst the GCR. Previous radiobiology studies with high energy proton beams using very thick absorbers (31) suggest that neutrons are ineffective in producing biological damage at high-energy (>100 MeV). This observation is readily predicted by the mean-free path of neutrons which is generally >10 cm for materials of interest. Because of the similarity of nuclear absorption cross sections, the secondary particles and target fragmentation spectrum produced by protons and neutrons of energies above a few hundred MeV are nearly identical. Thus high energy protons are biologically more effective compared to neutrons of the same energy per unit fluence because of their charge state. On the other hand, for very thick absorbers such as on the Martian surface or within a solar particle event storm shelter, low energy neutrons and concurrent depletion of HZE particles by the Martian atmosphere suggest a distinct reference field could be considered to simulate neutron spectra following the results of Kim et al. (13).

There are many areas of space radiation research which should continue to focus on track segment irradiation, including mechanistic studies of radiation quality and the development of data bases for improving radiation quality function models or dose-rate effects for cancer and non-cancer risks using multiple single particle species (MSPS) approaches. Studies of end-points such as chromosomal aberrations have already been made in space where biophysical models are shown to well produce measurements from astronauts (32). Prediction of the frequency of dicentric aberrations in lymphocytes (5.78 × 10^−3^) were compared to data from a Mir-18 crew member (6.4 + 2.0 × 10^−3^) and demonstrated good agreement (33). To repeat DNA damage experiments at a GCR simulator or other similar cell culture experiments is not recommended by the present authors because it would add little to reducing the uncertainties in risk estimates. Experiments with animals to reduce risk estimate uncertainties present other considerations as discussed next.

We have shown that the duration of a chronic GCR-simulated exposure to accurately reproduce the space situation should be several weeks or longer. However, a precise estimate will require understanding underlying mechanism for risk for the biological model considered. DNA damage processing is complete within a few hours for low LET radiation and 1–2 days for high LET radiation (34–36). For example, high LET radiation has been shown to have a preference for homologous recombination repair (37) due to the generation of short fragments (36) due to clustered DNA damage. Tissue responses including the TGFβ-Smad signaling pathway have been shown to control the DNA damage response (38–40) and can remain activated for a week or longer *in vivo*. Long relaxation times may be important for fluence-rate and exposure time considerations as described above. Other considerations are the turn-over times of different tissue types and the distinct mechanisms for targeted and non-targeted effects in cancer risk. In addition, slowly and rapidly dividing tissues could present distinct optimal chronic exposure times, and abscopal effects should be considered.

Animal experiments over several weeks present some unique challenges to particle accelerator experiments. Older studies of cancer risks with fission neutrons and gamma-rays supported by the Atomic Energy Commission and the Department of Energy (DoE) in the United States (41–43) were performed for as long as 60-days using specially designed irradiation facilities to house animals and were often restricted to an 8 h/day exposure regime to both facilitate animal use feasibility and represent conditions of radiation workers at nuclear reactors. The current exposure room at NSRL was not designed for long-term animal exposure. Astronauts are exposed in space on a 24 h/day cycle and the restriction to an 8 h/day exposure could introduce differences in biological responses due to circadian rhythm effects or unrepresentative DNA damage processing, cell cycle, or signal transduction cycles. Long-term exposure studies of CNS effects and interest in simulation of microgravity effects on radiation responses using the hind-limb suspension model in mouse have not been made in the past and it is not clear what experimental validation is needed prior to such studies under chronic irradiation conditions.

In considering CNS risks changes to cognition including memory can be through multiple mechanisms leading to changes to synapse (7). Synapse formation, stabilization, and decay have a fast actin dependent component (less than one day) and a slow plasticity dependent component (days to years) (32, 44). The average lifetime of synapses will vary in different regions of the brain and in comparison of mice or rats and humans. A black-box approach could consider varying the duration of the exposure, from a few days to a few weeks, to observe how CNS responses are changing with exposure time. However such experiments would involve large beam-time costs at current rates of >$6000 (US) per beam-time hr, present new experimental challenges to CNS radiobiology with animal models, and limit the number of studies to be performed because of their duration and time constraints at NSRL. Therefore if absent of an important scientific hypothesis, such studies should not be pursued. Development of the knowledge to predict CNS risks is favored over such black-box approaches.

There are also practical limitations to long-term exposures of a large number of mice or other small animals. The number of studies that can be performed at duration of month or longer is likely restricted to few per year, and the large costs of beam time that would result from such studies is a major obstacle. Several hundred highly constrained mice can be irradiated in a 60 × 60 cm^2^ beam configuration at NSRL for acute irradiation, however this approach is not practical for an exposure of several weeks including the requirement of replicate experiments for biological research. Risk model validation experiments are currently limited by the short-comings in available biological models of human risks, and the larger number of risks of interest (1–7). In addition the statistical errors in animal model data for track segment irradiations would likely complicate the interpretation of the outcome of a validation experiment with a GCR simulator.

It is of interest to explore new research areas that could be considered with a GCR simulation approach. One area of interest is a possible scientific hypothesis related to differences in biological responses for a mixed-field of particles of varying track structure due to synergistic interactions of particles of different radiation qualities. Very few low dose fractionation studies with protons and a single HZE particle species (45, 46) or fractionated HZE particles (47, 48) have been made and would be needed first to understand if synergistic effects are a valid concern. The few studies that have been made suggest that mixed radiation field synergistic effects, which violate the general principal of additivity used in radiation protection, will only occur if the mean inter-fraction times are <8 h (45–48). The validity of the additivity assumption used in radiation protection for the biological dose estimated for the endpoint of chromosomal aberrations was recently supported by a comparison of ISS crew-members participating in multiple ISS missions (49).

Another potential area of research with a GCR simulation is in the testing of biological countermeasures (BCM) with drug screening of panels of agents and different dosages for various space radiation risks. For BCM research, the matrix of risk types, radiation types and doses to be studied with different drug types and dosages in animals suggest the traditional approach to track segment irradiation may be at a very high cost for current space radiobiology efforts. On the other hand, the goals of BCM research are underdeveloped at this time. Acute risk BCM’s may not be needed because SPE organ doses are readily mitigated with shielding and alert dosimetry. Acute risk BCM’s may also be antagonistic to risks for late effects if they suppress apoptosis. Observations of low RBE’s for leukemia induction by HZE particles (50) suggest BCMs for this risk may not be needed except for an unexpected SPE exposures during extra-vehicular activity. For the risks of CNS and non-cancer late effects even less is known, including if dose thresholds for these risks will be exceeded for specific exploratory space missions, or how to extrapolate from animal models to human. At this time, BCM’s for solid cancer risks stand out as being a likely requirement for space missions. However, the mechanisms leading to the large RBE for HZE particle solid cancer and qualitative differences in tumor spectrum found in mice are poorly understood at this time (11). For mechanistic studies of GCR biological effects, the use of a GCR simulator would carry with it important concerns due to the complication of not knowing which spectral components produced an observed effect.

In summary, the development of our GCR simulation approach at NSRL is a promising long-term research goal especially for potential drug screening and BCM development approaches. However before such studies should be pursued, the mechanisms of space radiation risks and their underlying radiation quality and dose dependences need to be established. We find that the use of a GCR simulator to achieve uncertainty reduction in risk models suffers from several detrimental issues. Our analysis shows that for accelerator GCR simulations, a large percentage of cells will be hit with two or more particles in a simulated chronic exposure of a week or less and thus would not properly simulate the space condition. Therefore exposures of several weeks or longer will be needed to avoid such artifacts. This error will probably be higher for CNS risks compared to cancer risks because of the larger sizes of neuronal structures. GCR simulations for chronic times approaching 30 days are warranted to avoid any high dose-rate artifacts that will occur for shorter chronic exposures. There is no single “validation model” that can be suggested to measure risk and therefore is only through the totality of information from experimental and theoretical research that risk estimates are improved. Barring direct irradiation of humans at a GCR simulator, it is only through the development of more accurate biological models of space radiation risks and the underlying theoretical descriptions that these goals can be met, while the experimental complication of the use of mixed radiation fields would not likely facilitate this understanding. Near-term research focus should remain on these goals using track segment irradiations at low doses of HZE particles (<0.1 Gy) in support of the safety and well-being of astronauts participating in long-term space missions.

## Conflict of Interest Statement

The authors declare that the research was conducted in the absence of any commercial or financial relationships that could be construed as a potential conflict of interest.
